# Global *Escherichia coli* Sequence Type 131 Clade with *bla*_CTX-M-27_ Gene

**DOI:** 10.3201/eid2211.160519

**Published:** 2016-11

**Authors:** Yasufumi Matsumura, Johann D.D. Pitout, Ryota Gomi, Tomonari Matsuda, Taro Noguchi, Masaki Yamamoto, Gisele Peirano, Rebekah DeVinney, Patricia A. Bradford, Mary R. Motyl, Michio Tanaka, Miki Nagao, Shunji Takakura, Satoshi Ichiyama

**Affiliations:** Kyoto University Graduate School of Medicine, Kyoto, Japan (Y. Matsumura, T. Noguchi, M. Yamamoto, M. Tanaka, M. Nagao, S. Takakura, S. Ichiyama);; University of Calgary, Calgary, Alberta, Canada (J.D.D. Pitout, G. Peirano, R. DeVinney);; Kyoto University Graduate School of Engineering, Kyoto (R. Gomi, T. Matsuda);; AstraZeneca Pharmaceuticals LP, Waltham, Massachusetts, USA (P.A. Bradford);; Merck & Co., Inc., Rahway, New Jersey, USA (M.R. Motyl)

**Keywords:** β-lactamases, E. coli, multilocus sequence typing, MLST, molecular epidemiology, clones, high-throughput DNA sequencing, bacteria, Escherichia coli, bla gene, CTX-M-27, antimicrobial resistance

## Abstract

The *Escherichia coli* sequence type (ST) 131 C2/*H*30Rx clade with the *bla*_CTX-M-15_ gene had been most responsible for the global dissemination of extended-spectrum β-lactamase (ESBL)–producing *E. coli*. ST131 C1/*H*30R with *bla*_CTX-M-27_ emerged among ESBL-producing *E. coli* in Japan during the late 2000s. To investigate the possible expansion of a single clade_,_ we performed whole-genome sequencing for 43 Japan and 10 global ST131 isolates with *bla*_CTX-M-27_ (n = 16), *bla*_CTX-M-14_ (n = 16), *bla*_CTX-M-15_ (n = 13), and others (n = 8). We also included 8 ST131 genomes available in public databases. Core genome-based analysis of 61 isolates showed that ST131 with *bla*_CTX-M-27_ from 5 countries formed a distinct cluster within the C1/*H*30R clade, named C1-M27 clade. Accessory genome analysis identified a unique prophage-like region, supporting C1-M27 as a distinct clade. Our findings indicate that the increase of ESBL-producing *E. coli* in Japan is due mainly to emergence of the C1-M27 clade.

## Abstract 2

Increased extended-spectrum β-lactamase–producing *E. coli* in Japan resulted mainly from a clade containing this gene.

The global increase in resistance to the third-generation cephalosporins and fluoroquinolones among extraintestinal pathogenic *Escherichia coli* (ExPEC) is a public health concern because of the importance of these drugs in treating serious infections (*1*). The extended-spectrum β-lactamases (ESBLs), especially CTX-M types, contribute to third-generation cephalosporin resistance among ExPEC, and specific mutations in quinolone resistance–determining regions in *gyrA* and *parC* mainly contribute to fluoroquinolone resistance (*2*). The increase in resistance among ExPEC has resulted mainly from the recent expansion of a pandemic clonal group known as *E. coli* sequence type (ST) 131, which is usually multidrug resistant and is associated with CTX-M-15, the most prevalent β-lactamase among ESBL-producing ExPEC (*2*). ST131 harbors more virulence factors than other antimicrobial-resistant ExPEC and can cause severe infections (*2**,**3*).

Recent studies using whole-genome sequencing (WGS) analysis revealed that ST131 comprises different lineages or clades (*4**,**5*). Price et al. found a dominant fluoroquinolone-resistant lineage (named *H*30R) in North America that contains the *fimH* 30 allele and was associated with characteristic quinolone resistance–determining region mutations (*2**,**4*). ST131 with the *bla*_CTX-M-15_ gene formed a distinct cluster within the *H*30R lineage, referred to as the *H*30Rx clade (*4*). Petty et al. confirmed these findings using a collection of strains from 6 countries (*5*). In their study, *H*30R and *H*30Rx clades correspond to clade C and clade C2 (subset of clade C), respectively. The other clade C subset, clade C1, included ST131 isolates with different CTX-Ms than *bla*_CTX-M-15_.

Globally, the CTX-M-15–producing C2/*H*30Rx clade is mostly responsible for the pandemic of ExPEC with ESBLs (*2*), but in Japan, ExPEC with *bla*_CTX-M-15_ is rare despite the predominance of ST131 among ESBL-producing isolates (*6*). Before 2005, ST131 C1/*H*30R negative for Rx containing *bla*_CTX-M-14_ predominated among Japanese ST131 (*6*). In 2006, ST131 C1/*H*30R with *bla*_CTX-M-27_ was detected in Japan, and the numbers of this lineage escalated since 2010 and are responsible for the substantial increase of ESBL-producing ExPEC in Japan (*6*). Moreover, *bla*_CTX-M-27_ is confined to ST131, whereas other CTX-Ms, such as *bla*_CTX-M-14_ and *bla*_CTX-M-15_, are equally present among ST131 and non-ST131 *E. coli* isolates (*3*).

*bla*_CTX-M-27_ is an infrequent global *bla*_CTX-M_ allele that differs by only 1 nt from *bla*_CTX-M-14_, which results in 1 aa change at position 240 (*1**,**6*). ST131 with CTX-M-27 had previously been reported from other countries, such as Korea (isolation year 2008), China (2013–2014), Australia (2009–2010), Nepal (2013–2014), Cambodia (2004–2005), Israel (2008–2009), Czech Republic (2008–2011), Switzerland (2011), Spain (2012), France (2012), Portugal (2013–2014), Netherlands (2011), Canada (2005), and United States (2013) (*2**, **5**–**15*). Because of the rapid increase of CTX-M-27–producing ST131 in Japan since 2010 (*6*), we designed a study to characterize these isolates using WGS techniques.

## Materials and Methods

### Bacterial Isolates

We selected 43 nonduplicate ST131 clinical isolates collected from 2 multicenter surveillance programs in Japan for WGS to represent 3 major ESBL-producing ST131 (CTX-M-27–producing *H*30R, 13 isolates; CTX-M-14–producing *H*30R, 9 isolates; CTX-M-15–producing *H*30Rx, 11 isolates) and other ST131 (CTX-M-14+CTX-M-15–producing *H*30Rx, 2 isolates; CTX-M-14–producing *H*30Rx, 1 isolate; CTX-M-14–producing *H*22, 1 isolate; CTX-M-2–producing *H*22, 1 isolate; TEM-producing *H*30, 2 isolates; non–ESBL-producing *H*30R, 3 isolates) in Japan (*6*) (Table). One of the surveillance programs collected ESBL-producing *E. coli* isolates during 2001–2010 at 10 acute-care hospitals in the Kyoto and Shiga prefectures of Japan (*6*); the other program collected all *E. coli* isolates during December 2014 at 10 acute-care hospitals in the 5 prefectures in central Japan. ST131 isolates were identified by PCR specific for *mdh* and *gyrB* alleles, O25b or O16 *rfb* variants, *fimH* allele, and *H*30Rx status (*6*). The selection process of the Japanese ST131 ensured equal representation by geographic location, specimen type, and date of isolation.

**Table:** *Escherichia coli* ST131 isolates included in study of the global expansion of a single clade*

Type of ESBL	Country/prefecture of isolation (no. isolates; year)			
	*H*30			*H*22, n = 2
	*H*30R		*H*30, n = 2	
	*H*30R, n = 39	*H*30Rx, n = 18		
CTX-M-27, n = 21	Japan/Kyoto, Shiga, Aichi (13; 2004–2014), Australia (3; 2009, 2010),† United States (2; 2013, 2014),‡ Canada (1; 2008), Thailand (1; 2013), Vietnam (1; 2011)			
CTX-M-14, n = 17	Japan/Kyoto, Shiga, Hyogo (9; 2002–2014), Canada (2; 2005, 2009),† France (1; 2008), New Zealand (1; 2010), South Africa (1; 2008), United States (1; 2008)	Japan/Kyoto (1; 2009)		Japan/Kyoto (1; 2007)
CTX-M-15, n = 15		Japan/Kyoto, Shiga, Osaka (11; 2006–2014), Canada (2; 2009), UK (1; 2005),§ United States (1; 2008)¶		
CTX-M-14 and CTX-M-15, n = 2		Japan/Kyoto (2; 2010, 2014)		
CTX-M-2, n = 1				Japan/Kyoto (1; 2004)
TEM, n = 2			Japan/Kyoto (2; 2005, 2009)	
Negative, n = 3	Japan/Shiga, Hyogo, Osaka (3; 2014)			

*ESBL, extended-spectrum β-lactamase; ST, sequence type. †Raw reads were downloaded from European Nucleotide Archive (accession no. ERA118286) for 3 isolates from Australia (S100EC, S107EC, S108EC) and 1 isolate from Canada (S135EC). ‡MRSN17749 draft genome (GenBank assembly accession no. GCA_000770275.1) and IEH71520 draft genome (GenBank assembly accession no. GCA_000681435.1). §EC958 complete genome (GenBank assembly accession no. GCA_000285655.3). ¶JJ1886 complete genome (GenBank assembly accession no. GCA_000493755.1).

In addition to isolates from Japan, we obtained 10 CTX-M–producing ST131 isolates from global collections that previously had been characterized by multilocus sequence typing (MLST) (Table; Technical Appendix Table 1). We selected all of the CTX-M-27 producers, 1 CTX-M-14 producer per country, and 2 CTX-M-15 producers. We also sought public databases for ST131 *H*30 and included sequence data for 8 isolates from countries other than Japan: CTX-M-27 producers (3 raw reads, 2 draft genomes); CTX-M-14 producer (1 raw read); and CTX-M-15–producing C2/*H*30Rx (2 complete genomes) (Table; Technical Appendix Table 1) (*5**,**9**,**16**–**18*).

### WGS

We used the Nextera XT DNA sample preparation kit (Illumina, San Diego, CA, USA) to prepare libraries for sequencing. Samples were multiplexed and sequenced on an Illumina MiSeq for 600 cycles (300-bp paired-end) or NextSeq500 for 300 cycles (151-bp paired-end). The ST131 genomes were sequenced at an average depth of 44.03 (SD ± 14.70) and an average coverage of 97.73% (SD ± 0.93%) using the 5,109,767-bp EC958 chromosome as previously described (*16*).

### Core Genome Analysis

We used a core genome single-nucleotide polymorphism (SNP)–based approach to create a phylogenetic tree. We identified SNPs using raw read mapping followed by duplicate read removal, realignment, quality score recalibration, and variant filtering (Technical Appendix). Reads from 53 isolates sequenced in this study and 4 isolates (S100EC, S107EC, S108EC, and S135EC) (*5*) were aligned against a reference genome of EC958, and SNPs were called. The remaining 4 draft or complete genomes underwent whole-genome alignment against EC958 to make EC958-like pseudo-chromosomes that contained only SNPs. The SNP-only core genome was identified as the blocks of >500 bp common to all 61 study isolates to ensure that each block represented a common segment from good alignment in each isolate and that the block had enough length to enable identification (*5*). A maximum-likelihood tree was built using RAxML (*19*). A recombination-free tree was also build by excluding recombination sites identified using a Bayesian analysis software BRATNextGen (*20*).

### Comparative Genomic Analysis

To define presence of genes and their alleles, we used SRST2 with trimmed reads or BLAST+ (executables [http://blast.ncbi.nlm.nih.gov/]) with assembled draft genomes and following databases or typing schemes: ResFinder antimicrobial resistance gene database, VFDB and VirulenceFinder virulence gene databases, serotypeFinder O:H typing database, PlasmidFinder plasmid replicon database, MLST (http://mlst.ucc.ie/mlst/dbs/Ecoli), plasmid MLST, *fimH* typing, *gyrA*/*parC* typing, ST131 virotyping, and detection of *H*30Rx-specific *ybbW* SNPs, plasmid addiction systems, and *bla*_CTX-M_ genetic environment (online Technical Appendix). We used pangenome analysis to identify clade specific segments among draft or complete genomes. BRIG was used to visualize similarity of genomes to ST131 genomic islands (*16*) and to the ST131 reference plasmid pEC958 (*21*).

### Statistical Analysis and Sequence Data Accession Numbers

We compared categorical variables using Fisher exact test. A p value <0.05 was considered statistically significant. We conducted our statistical analysis using Stata, version 13.1 (StataCorp, College Station, TX, USA). The sequences were deposited in the DDBJ Sequence Read Archive database (accession no. DRA004266 and DRA004267).

## Results

### Bacterial Isolates

The study comprised 60 clinical and 1 environmental ST131 isolates (Table; Technical Appendix Table 1). We confirmed the types of β-lactamase genes, ST131 status, *fimH* allele numbers, and *H*30Rx status using draft genomes.

### Core Genome SNP-based Phylogenetic Tree

**Figure 1:**
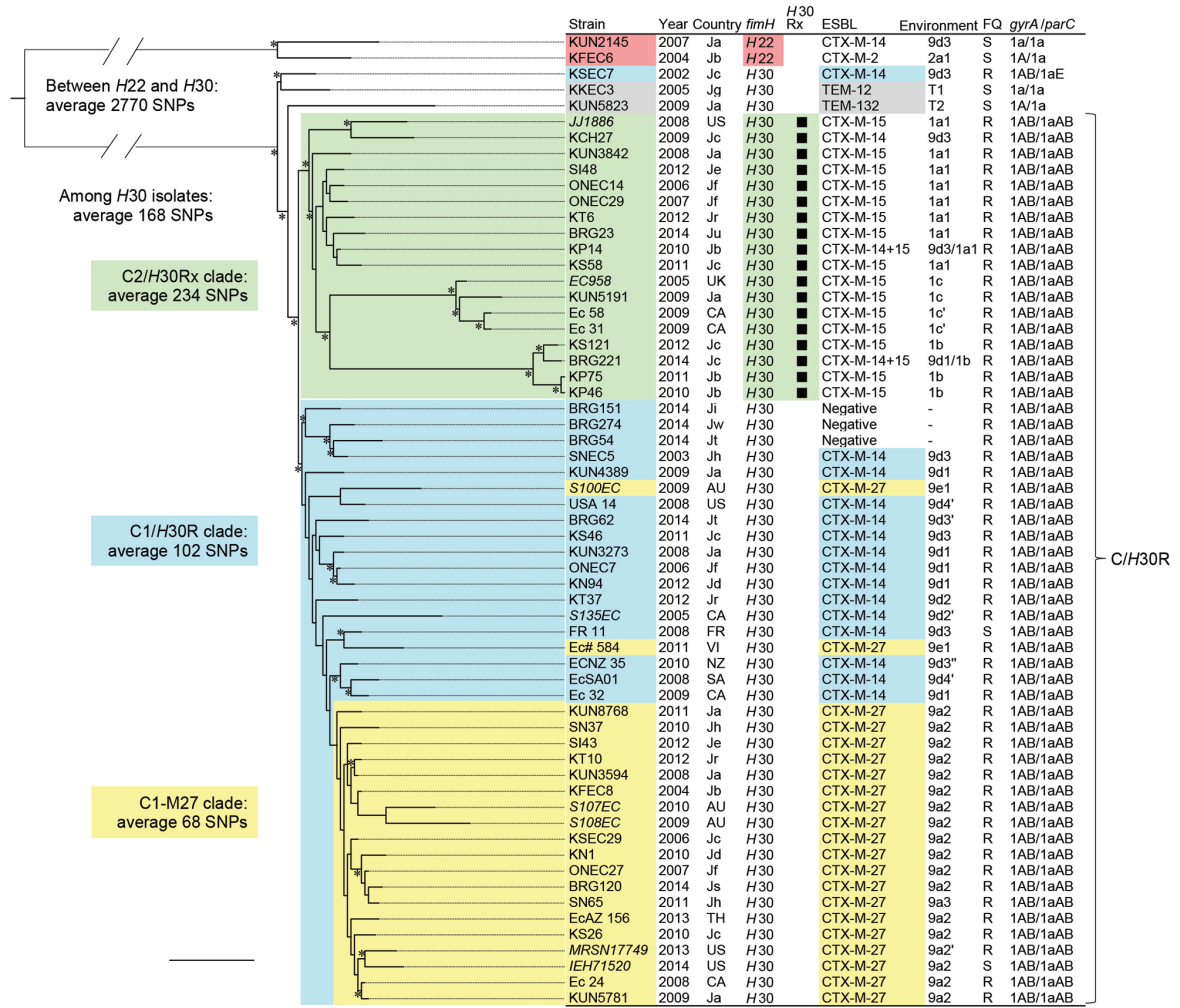
Core genome single-nucleotide polymorphism (SNP)–based phylogenetic tree of *Escherichia coli* sequence type (ST) 131 isolates. This maximum-likelihood phylogram is based on a 4,086,650-bp core genome and a total of 5,280 SNPs. The tree is rooted by using the outgroup *H*22 isolates, and asterisks indicate bootstrap support >90% from 100 replicates. Strains that had previously been sequenced are in *bold*. The Country columns indicate places of isolation: Ja to Jw, Japan (a to w indicates hospitals); AU, Australia; CA Canada; FR, France; NZ, New Zealand; SA, South Africa; TH, Thailand; UK, United Kingdom; US, United States; VI, Vietnam. Environment column shows a type of genetic environment of ESBL genes (Technical Appendix Table 2). FQ columns indicate ciprofloxacin susceptibilities (S, susceptible; R, resistant). KSEC7 had a *parC* 1aE allele including G250A (S80K) mutation in addition to a 1a allele. The ciprofloxacin-resistant C/*H*30R cluster comprised the C2/*H*30Rx and C1/*H*30R clades. All of the *H*30Rx isolates belonged to the C2/*H*30Rx clade. The C1/*H*30R clade included CTX-M-14–producing *H*30R, non–ESBL-producing *H*30R, and CTX-M-27–producing *H*30R isolates. CTX-M-27–producing isolates belonged to the C1-M27 clade within the C1/*H*30R clade except 2 isolates (S100EC and EC# 584). The bootstrap value for the root of the C1-M27 clade was 64%. An average of 68 SNPs was found among the C1-M27 clade, whereas an average of 158 SNPs was found between the C1-M27 clade and 2 non–C1-M27 clade isolates with *bla*_CTX-M-27_. Scale bar indicates 100 SNPs.

We identified a 4,086,650-bp core genome that included 5,280 SNPs by mapping and alignment of the 61 study isolates to EC958 (Figure 1). The ciprofloxacin-resistant isolates with *gyrA* 1AB and *parC* 1aAB alleles formed the C/*H*30R cluster that comprised the C2/*H*30Rx and C1/*H*30R clades. The C2/*H*30Rx clade included isolates with *bla*_CTX-M-15_ (n = 15) and *bla*_CTX-M-14_ (n = 1) and isolates with both *bla*_CTX-M-15_ and *bla*_CTX-M-14_ (n = 2) (Figure 1). The C1/*H*30R clade included isolates with *bla*_CTX-M-27_ (n = 21) and *bla*_CTX-M-14_ (n = 14) and isolates without ESBLs (n = 3) (Figure 1). Within the C1/*H*30R clade, 19 of 21 CTX-M-27–producing isolates clustered into a distinct group, named the C1-M27 clade (Figure 1). *E. coli* ST131 C1-M27 comprised isolates from Japan (n = 13; isolation years 2004–2014), Australia (n = 2; 2009–2010), United States (n = 2; 2013–2014), Canada (n = 1, 2008), and Thailand (n = 1, 2013).

Analysis of the core genome showed that 79 segments (i.e., 304,782 bp, including 3,453 SNPs) were associated with recombination sites (Technical Appendix Figure 1). This finding suggests that recombinant segments contained 65% of SNPs with subsequent higher frequency of SNPs compared with nonrecombinant regions (average 11 vs. 0.48 SNPs/kb, respectively). The phylogenetic tree created without recombination sites showed the same results as the phylogenetic tree obtained with recombination sites (Technical Appendix Figure 2). In addition to the core genome–based phylogeny with or without recombination sites, the C1-M27 clade was defined by a unique accessory genome of the M27PP1.

### The C1-M27 Clade–Specific Region

**Figure 2:**
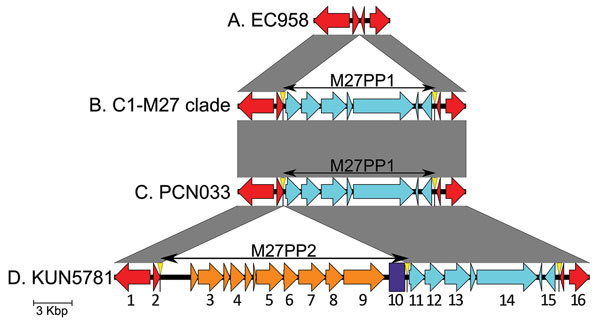
Genetic environments of the C1-M27 clade–specific region of *Escherichia coli*. All isolates other than the C1-M27 clade isolates had the type A structure in their chromosome (red arrows; gene locus tags shown in the bottom are annotated according to EC958). The C1-M27 clade isolates except 2 isolates (KUN5781 and Ec 24) had the type B structure. A 11,894-bp region (M27PP1; predicted genes shown in light blue arrows) is inserted into the type A structure creating the 7-bp direct repeat (CCGTTCT; yellow triangle). The inserted sequence M27PP1 is identical to a prophage-like genomic island in *E. coli* PCN033 chromosome (GenBank accession no. CP006632), which had the similar flanking structure (structure C, 98.8% similarity). M27PP1 included phage-like integrase and recombinase. The identical M27PP1 sequence was found in all of the C1-M27 isolates with the use of additional Sanger sequencing. Only the draft genome of IEH71520 had 98.7% coverage to the M27PP1 sequence because of contig discontinuity. KUN5781 and Ec 24 had the type D structure, of which an additional 19,352-bp region (M27PP2) is inserted into the type B structure by creating the same 7-bp direct repeat (yellow triangle). The M27PP2 includes a total of 15,555-bp region (genes shown in orange arrows), which was 88.9% similar to a prophage-like region in γ proteobacterium HdN1 chromosome (GenBank accession no. FP929140) and a following 1,221-bp region is 99.8% similar to IS*Sen4* (purple box). Code to gene locus tags: 1, 958RS23365; 2, 958RS23370; 3, HDN1F03950; 4, HDN1F03970; 5, HDN1F04000; 6, HDN1F04010; 7, HDN1F04020; 8, HDN1F04030; 9, HDN1F04040; 10, ISSen4; 11, 033RS22420; 12, 033RS22425; 13, 033RS22430; 14, 033RS22440; 15, 033RS22450; 16, 958RS23380.

The pangenome analysis of genomes from all the isolates identified an 11,894-bp region named M27PP1 that was specific to all the isolates from the C1-M27 clade. Further analysis using the BLAST database and Sanger sequencing for gap filling showed that this region was identical to a prophage-like genomic island (GenBank accession no. CP006632) in *E. coli* PCN033 that belonged to phylogenetic group D and was isolated from a pig in China. The BLAST database also identified 2 similar sequences (i.e., 99.9% homology): A CMY-2 containing plasmid pEQ011 (GenBank accession no. NC_023315) in an *E. coli* isolate from a horse in Ireland (*22*) and a multidrug-resistant plasmid pSD853_88 (GenBank accession no. JF267652) found in a bovine *Salmonella enterica* isolate in the United States. M27PP1 was inserted into chromosome creating a 7-bp direct repeat region (Figure 2). PCN033 had the same flanking structure as the M27PP1, whereas the 2 plasmids (pEQ011, pSD853_89) contained only a 44-bp similar segment at 5′ side and other parts of these plasmids were not found in the C1-M27 clade isolates.

Two *E. coli* ST131 C1-M27 isolates (i.e., KUN5781 and Ec 24) had an additional insertion region of 19,352 bp, named M27PP2, situated upstream of M27PP1. M27PP2 was accommodated within the same 7-bp direct repeat region (Figure 2). M27PP2 included a 15,555-bp region that showed 88.9% homology to a prophage-like sequence in the chromosome of the γ proteobacterium HdN1 (GenBank accession no. FP929140) and 99.8% homology to the insertion element IS*Sen4*.

### Genomic Comparison of the ST131 Genomic Islands and Virulence Genes

**Figure 3:**
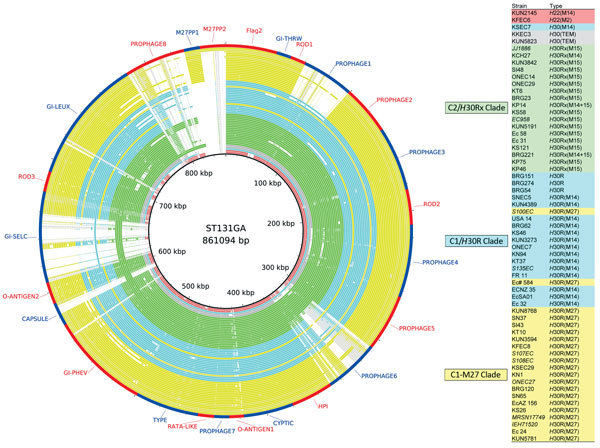
Genome similarities to the *Escherichia coli* sequence type (ST) 131 genomic islands and the C1-M27 clade–specific region. Rings drawn by BRIG show the presence of these regions. Colored segments indicate >90% similarity and gray segments indicate >70% similarity by BLAST comparison between the regions of interest and each genome. Extended-spectrum β-lactamase types are indicated in parentheses of Type column. The regions from Flag2 to GI-*lueX* were found in EC958, the prophage 8 region was found in JJ1886, and the M27PP1 and M27PP2 were found as the C1-M27 clade–specific regions in this study. Prophage 6, capsule, GI-*selC*, and prophage 8 regions were present in some C2/*H*30Rx isolates but were absent in C1/*H*30R isolates.

The sequences of the study isolates were similar to the ST131 genomic islands in EC958 and JJ1886 (a CTX-M-15–producing C2/*H*30Rx strain obtained in the United States from a patient with fatal urosepsis) (Figure 3) (*17*). The C1-M27 clade isolates lacked the prophage 1 region present in EC958 (Figure 3). This prophage 1 region, specific for ST131, was present among the non–C1-M27 ST131 isolates in this study, except for BRG23 and EcSA01. The presence of ExPEC-associated virulence genes is shown in Technical Appendix Figure 3. The *senB* enterotoxin gene was more common in C1/*H*30R (than in C2/*H*30Rx). No significant differences existed in the distribution of virulence genes between *E. coli* ST131 C1-M27 and other isolates.

### Plasmid Replicons, Addiction Systems, and Antimicrobial Drug Resistance Genes

We compared the study isolates with pEC958, the plasmid present in EC958 that carries *bla*_CTX-M-15_ (Technical Appendix Figure 4). The C1-M27 clade lacked the first part of the transfer region (*tra*) present in pEC958. Some regions common to both C2/*H*30Rx and C1/*H*30R clades were present in pEC958. The C1/*H*30R clade producing CTX-M-27 or CTX-M-14 (including C1-M27) contained mostly F1:A2:B20 replicons, whereas the C2/*H*30Rx clade producing CTX-M-15 contained mainly F2:A1:B- replicons (Technical Appendix Figure 5). The C1-M27 clade was negative for Tn*2* containing *bla*_TEM-1_. Two C1-M27 isolates from Thailand and the United States were also positive for *bla*_NDM-1_ (Technical Appendix Figure 5).

## Discussion

A previously unreported clade named C1-M27 within C1/*H*30R clade is responsible for the epidemic of ESBL-producing ExPEC in Japan and has already been disseminated to 5 countries on 3 continents. ST131 containing *bla*_CTX-M-27_ responsible for human infections has been reported from various continents (*2*) and is especially common among ESBL-producing ExPEC in certain countries, such as Israel, the Czech Republic, and Switzerland (*2**,**13**,**14*). CTX-M-27–producing ST131 also is present among nonclinical and nonhuman *E. coli* isolates, including fecal specimens of healthy children attending day care centers in France; fecal specimens of healthy adults in China, Portugal, and the Netherlands; samples from sick dogs and cats in Japan; samples from water birds from central Europe and Swiss rivers and lakes; and samples of well water from China (*2**,**10**,**11**,**15**,**23**–**25*). The most common ESBL among *E. coli* ST131 in nonhuman samples is CTX-M-27 (*2**,**23**–**25*). ST131 with *bla*_CTXM-15_ is rare among animal and environmental *E. coli* isolates (*26*). Our analysis of IEH71520, an *E. coli* isolate from vacuum cleaner dust in the United States (*15*), showed that this ST131 isolate belong to the C1-M27 clade. The C1-M27 clade is likely to be present among animal and environmental ST131, and such isolates might act as a hidden reservoir for the introduction of ST131 containing *bla*_CTX-M-27_ into human medicine.

*E. coli* ST131 C1-M27 had an additional, unique prophage-like region (M27PP1) within its chromosome, lacked the prophage 1 genomic island previously identified in ST131 C2/*H*30Rx, and were negative for the transposon Tn*2* containing *bla*_TEM-1_ (Figure 3; Technical Appendix Figure 5). The direct flanking repeat sequences surrounding M27PP1 suggest that this region was introduced into *E. coli* ST131 C1/*H*30R with *bla*_CTX-M-27_ by a recombination event that was then followed by the clonal expansion of the C1-M27 clade.

Recent studies focusing on evolutionary history of ST131 suggested that C1/*H*30R and C2/*H*30Rx clades emerged ≈30 years ago, after their acquisition of *gyrA*-1AB and *parC*-1aAB alleles from C0/*H*30 (non-R) clade (*27**,**28*). The phylogeny and smaller numbers of SNPs in the C1-M27 clade (Figure 1) suggest that this clade was recently diverged from the C1/*H*30R. In the time-scaled phylogeny presented by Stoesser et al. (*27*), a cluster that included 6 CTX-M-27–producing isolates from Cambodia, Thailand, and Laos in 2007–2011 was present within the C1/*H*30R clade. This cluster, supposed to be the C1-M27 clade, diverged in the early 2000s, supporting our hypothesis.

CTX-M-27–producing ST131 that belongs to the *H*41 lineage previously had been described from Japan (*6*) and China (*15*). The characterization of the Japanese ST131 *H*41 isolates showed different genetic structures flanking the *bla*_CTX-M-27_ from those structures present in *E. coli* ST131 *H*30R (*6*). The flanking regions previously characterized in ST131 *H*41 were identical to the flanking regions in ST131 non–C1-M27 from this study. It seems there are 2 types of structures flanking the *bla*_CTX-M-27_ among *E. coli* ST131; 1 type is confined to clade C1-M27 (i.e., 208 bp of ΔIS*Ecp1* upstream and ΔIS*903D* downstream), whereas another structure (i.e., 388 bp of ΔIS*Ecp1* upstream and full IS*903D* downstream) is distributed among non–C1-M27 isolates, including ST131 *H*41 (*6*). Therefore, ST131 *H*41, through horizontal transfer of *bla*_CTX-M-27_, is unlikely to have played a substantial role in the emergence of the C1-M27 clade.

Two ST131 isolates with *bla*_CTX-M-27_ from Australia and Vietnam did not belong to the C1-M27 clade (Figure 1). These 2 isolates differ from the C1-M27 clade in that their core genomes had more SNPs (158 vs. 68), contained the prophage 1 ST131-specific region, and lacked the M27PP1 and M27PP2 elements. Moreover, the genetic environment surrounding the *bla*_CTX-M-27_ differed from *E. coli* ST131 C1-M27 (as described previously). The isolate from Vietnam lacked *mph*(A)*-mrx-mphR*, *tetR-tet*(A), *sul2-strA-strB*, and In*54* resistance genes, compared with the C1-M27 clade (Technical Appendix Figure 5). These differences indicate that some ST131 isolates might acquire *bla*_CTX-M-27_ independently from the C1-M27 clade.

Our study has several limitations. Most isolates originated from Japan. However, we were able to include ST131 C1-M27 isolates from 5 countries on 3 continents and C1/*H*30R isolates producing CTX-M-14 or CTX-M-15 from 6 countries on 4 continents. Another limitation was that we were able to obtain only 1environmental ST131 isolate with *bla*_CTX-M-27_ (IEH71520). Future studies that include environmental isolates will provide additional insights into molecular epidemiology and evolutionary history of the C1-M27 clade. We could not analyze plasmid contents of *bla*_CTX-M-27_ because *bla*_CTX-M-27_–containing contigs were too short. The sequencing of plasmids that contain *bla*_CTX-M-27_ obtained from various ST131 clades (including the C1-M27 clade) should also be undertaken.

In conclusion, we showed that the recent increase in ESBL-producing *E. coli* from Japan resulted from emergence of a ST131 C1/*H*30R subclade with *bla*_CTX-M-27_. This clade, named C1-M27, had unique genomic characteristics and was present in ST131 from Thailand, Australia, Canada, and the United States. Our findings suggest that the C1-M27 clade is contributing to the global success of ST131. *E. coli* ST131 C1-M27 poses a major new public health threat because of its global distribution and association with the very dominant C/*H*30 lineage. We urgently need rapid cost-effective detection methods for *E. coli* ST131 C1-M27 and well-designed epidemiologic and molecular studies to understand the dynamics of transmission, risk factors, and reservoirs for ST131 C1-M27. These efforts will provide insight into the emergence and spread of this multidrug-resistant clade that will lead to information essential for preventing the spread of ST131.

Technical Appendix Supplementary methods, strain information, mapping, assembly statistics, genetic structures recombinant regions and other details of *Escherichia coli* sequence type 131 isolates.
