# Supplementary material for: Global Escherichia coli Sequence Type 131 Clade with blaCTX-M-27 Gene
Source: Emerg Infect Dis. 2016 Nov;22(11):1900–7. doi: 10.3201/eid2211.160519 (PMC5088012; doi:10.3201/eid2211.160519)
Supplement: Technical Appendix — Supplementary methods, strain information, mapping, assembly statistics, genetic structures recombinant regions and other details of Escherichia coli sequence type 131 isolates. [file 16-0519-Techapp-s1.pdf]

Article DOI: http://dx.doi.org/10.3201/eid2211.160519

Global *Escherichia coli* Sequence Type 131 Clade with *bla*$_{CTX-M-27}$ Genes

Technical Appendix

Supplementary Methods

We used a core genome single-nucleotide polymorphism (SNP)–based approach to create a phylogenetic tree using the current standard procedure (*1*). SNPs were identified using raw read mapping followed by duplicate read removal, realignment, quality score recalibration, and variant filtering (*2*).

Core Genome Analysis

Reads from 53 isolates sequenced in this study and 4 isolates (S100EC, S107EC, S108EC, and S135EC) (*3*) underwent quality trimming using ERNE-FILTER (*4*). Trimmed reads were aligned against a reference genome of EC958 using Burrows-Wheeler Aligner (*5*). SNPs were called by using GATK Best Practices workflow (*6*) and SAMtools (coverage >10 and Phred-score >20) (*7*). The remaining 4 draft or complete genomes were aligned against EC958 by using ProgressiveMauve (*8*) to make EC958-like pseudo-chromosomes that contained only SNPs. The SNP-only core genome was identified as the common blocks of >500 bp to all 61 study isolates by using in-house Perl script. A maximum-likelihood tree was build using RAxML with GTR GAMMA substitution model and 100 rapid bootstrap replicates (*9*). We also separately analyzed the phylogeny of the sequence type (ST) 131 isolates excluding recombination sites. Bacterial recombination occurs more frequently than spontaneous mutations, and a phylogenetic tree that includes recombination sites could potentially distort phylogenetic inference (*10*), although this is not universally accepted as dogma (*11*). A recombination-free tree was also build by excluding recombination sites identified using a Bayesian analysis software BRATNextGen (*12*). A cutoff in the proportion of shared ancestry tree was chosen to enable separation of clades found in core genome-based tree. Twenty iterations of hidden Markov model parameter estimation were performed, and 100 permutations resampling was performed to determine the statistically significant recombination segments ($p<0.05$).

Genome Assembly

Trimmed reads were assembled by using Velvet and VelvetOptimizer (*13*) with k-mer values ranging from 31 to 73. The best assembly results in terms of the highest N50 value of each isolate underwent refinement of draft genomes using PAGIT (*14*).

Comparative Genomic Analysis

To define presence of genes and their alleles, we mapped trimmed reads to reference genes using SRST2 (*15*) and used BLAST+ (*16*) for draft or complete genomes. We used the following databases or typing schemes: ResFinder antimicrobial resistance gene database (*17*) VFDB (*18*) and VirulenceFinder (*19*) virulence gene databases, serotypeFinder O:H typing database (*20*), PlasmidFinder plasmid replicon database (*21*), MLST (http://mlst.ucc.ie/mlst/dbs/Ecoli), plasmid MLST (*21*), *fimH* typing (*22*), *gyrA*/*parC* typing (*22*), ST131 virotyping (*23*), and detection of *H*30Rx-specific *ybbW* SNP (*24*), plasmid addiction systems (*25*), and $bla_{\text{CTX-M}}$ genetic environment (*26*). Gegenees (*27*) was used to identify clade-specific segments among draft or complete genomes and visualized with EasyFig (*28*). BRIG (*29*) was used to visualize similarity of genomes to ST131 genomic islands (*30*) and to the ST131 reference plasmid pEC958 (*31*).

References

1. Robinson ER, Walker TM, Pallen MJ. Genomics and outbreak investigation: from sequence to consequence. Genome Med. 2013;5:36. PubMed http://dx.doi.org/10.1186/gm440

2. Olson ND, Lund SP, Colman RE, Foster JT, Sahl JW, Schupp JM, et al. Best practices for evaluating single nucleotide variant calling methods for microbial genomics. Front Genet. 2015;6:235. PubMed http://dx.doi.org/10.3389/fgene.2015.00235

3. Petty NK, Ben Zakour NL, Stanton-Cook M, Skippington E, Totsika M, Forde BM, et al. Global dissemination of a multidrug resistant *Escherichia coli* clone. Proc Natl Acad Sci U S A. 2014;111:5694–9. PubMed http://dx.doi.org/10.1073/pnas.1322678111

4. Del Fabbro C, Scalabrin S, Morgante M, Giorgi FM. An extensive evaluation of read trimming effects on Illumina NGS data analysis. PLoS ONE. 2013;8:e85024. PubMed http://dx.doi.org/10.1371/journal.pone.0085024

5. Li H, Durbin R. Fast and accurate short read alignment with Burrows-Wheeler transform. Bioinformatics. 2009;25:1754–60. PubMed http://dx.doi.org/10.1093/bioinformatics/btp324

6. McKenna A, Hanna M, Banks E, Sivachenko A, Cibulskis K, Kernytsky A, et al. The Genome Analysis Toolkit: a MapReduce framework for analyzing next-generation DNA sequencing data. Genome Res. 2010;20:1297–303. PubMed http://dx.doi.org/10.1101/gr.107524.110

7. Li H, Handsaker B, Wysoker A, Fennell T, Ruan J, Homer N, et al. The Sequence Alignment/Map format and SAMtools. Bioinformatics. 2009;25:2078–9. PubMed http://dx.doi.org/10.1093/bioinformatics/btp352

8. Darling AE, Mau B, Perna NT. progressiveMauve: multiple genome alignment with gene gain, loss and rearrangement. PLoS ONE. 2010;5:e11147. PubMed http://dx.doi.org/10.1371/journal.pone.0011147

9. Stamatakis A. RAxML version 8: a tool for phylogenetic analysis and post-analysis of large phylogenies. Bioinformatics. 2014;30:1312–3. PubMed http://dx.doi.org/10.1093/bioinformatics/btu033

10. Rannala B, Yang Z. Phylogenetic inference using whole genomes. Annu Rev Genomics Hum Genet. 2008;9:217–31. PubMed http://dx.doi.org/10.1146/annurev.genom.9.081307.164407

11. Hedge J, Wilson DJ. Bacterial phylogenetic reconstruction from whole genomes is robust to recombination but demographic inference is not. MBio. 2014;5:e02158. PubMed

12. Marttinen P, Hanage WP, Croucher NJ, Connor TR, Harris SR, Bentley SD, et al. Detection of recombination events in bacterial genomes from large population samples. Nucleic Acids Res. 2012;40:e6. PubMed http://dx.doi.org/10.1093/nar/gkr928

13. Zerbino DR. Using the Velvet de novo assembler for short-read sequencing technologies. Curr Protoc Bioinformatics. 2010 Sep; Chapter 11:Unit 11.5. http://dx.doi.org/10.1002/0471250953.bi1105s31 **PMID: 20836074**

14. Swain MT, Tsai IJ, Assefa SA, Newbold C, Berriman M, Otto TD. A post-assembly genome-improvement toolkit (PAGIT) to obtain annotated genomes from contigs. Nat Protoc. 2012;7:1260–84. PubMed http://dx.doi.org/10.1038/nprot.2012.068

15. Inouye M, Dashnow H, Raven LA, Schultz MB, Pope BJ, Tomita T, et al. SRST2: rapid genomic surveillance for public health and hospital microbiology labs. Genome Med. 2014;6:90. PubMed http://dx.doi.org/10.1186/s13073-014-0090-6

16. Camacho C, Coulouris G, Avagyan V, Ma N, Papadopoulos J, Bealer K, et al. BLAST+: architecture and applications. BMC Bioinformatics. 2009;10:421. PubMed http://dx.doi.org/10.1186/1471-2105-10-421

17. Zankari E, Hasman H, Cosentino S, Vestergaard M, Rasmussen S, Lund O, et al. Identification of acquired antimicrobial resistance genes. J Antimicrob Chemother. 2012;67:2640–4. PubMed http://dx.doi.org/10.1093/jac/dks261

18. Chen L, Xiong Z, Sun L, Yang J, Jin Q. VFDB 2012 update: toward the genetic diversity and molecular evolution of bacterial virulence factors. Nucleic Acids Res. 2012;40:D641–5. PubMed http://dx.doi.org/10.1093/nar/gkr989

19. Joensen KG, Scheutz F, Lund O, Hasman H, Kaas RS, Nielsen EM, et al. Real-time whole-genome sequencing for routine typing, surveillance, and outbreak detection of verotoxigenic *Escherichia coli*. J Clin Microbiol. 2014;52:1501–10. PubMed http://dx.doi.org/10.1128/JCM.03617-13

20. Joensen KG, Tetzschner AM, Iguchi A, Aarestrup FM, Scheutz F. Rapid and easy in silico serotyping of *Escherichia coli* isolates by use of whole-genome sequencing data. J Clin Microbiol. 2015;53:2410–26. PubMed http://dx.doi.org/10.1128/JCM.00008-15

21. Carattoli A, Zankari E, García-Fernández A, Voldby Larsen M, Lund O, Villa L, et al. In silico detection and typing of plasmids using PlasmidFinder and plasmid multilocus sequence typing. Antimicrob Agents Chemother. 2014;58:3895–903. PubMed http://dx.doi.org/10.1128/AAC.02412-14

22. Johnson JR, Tchesnokova V, Johnston B, Clabots C, Roberts PL, Billig M, et al. Abrupt emergence of a single dominant multidrug-resistant strain of *Escherichia coli*. J Infect Dis. 2013;207:919–28. PubMed http://dx.doi.org/10.1093/infdis/jis933

23. Blanco J, Mora A, Mamani R, López C, Blanco M, Dahbi G, et al. Four main virotypes among extended-spectrum-β-lactamase–producing isolates of *Escherichia coli* O25b:H4–B2-ST131: bacterial, epidemiological, and clinical characteristics. J Clin Microbiol. 2013;51:3358–67. PubMed http://dx.doi.org/10.1128/JCM.01555-13

24. Banerjee R, Robicsek A, Kuskowski MA, Porter S, Johnston BD, Sokurenko E, et al. Molecular epidemiology of *Escherichia coli* sequence type 131 and Its H30 and H30-Rx subclones among extended-spectrum-β-lactamase-positive and -negative *E. coli* clinical isolates from the Chicago region, 2007 to 2010. Antimicrob Agents Chemother. 2013;57:6385–8. PubMed http://dx.doi.org/10.1128/AAC.01604-13

25. Mnif B, Vimont S, Boyd A, Bourit E, Picard B, Branger C, et al. Molecular characterization of addiction systems of plasmids encoding extended-spectrum beta-lactamases in *Escherichia coli*. J Antimicrob Chemother. 2010;65:1599–603. PubMed http://dx.doi.org/10.1093/jac/dkq181

26. Matsumura Y, Johnson JR, Yamamoto M, Nagao M, Tanaka M, Takakura S, et al. CTX-M-27– and CTX-M-14–producing, ciprofloxacin-resistant *Escherichia coli* of the *H*30 subclonal group within ST131 drive a Japanese regional ESBL epidemic. J Antimicrob Chemother. 2015;70:1639–49. PubMed

27. Agren J, Sundström A, Håfström T, Segerman B. Gegenees: fragmented alignment of multiple genomes for determining phylogenomic distances and genetic signatures unique for specified target groups. PLoS ONE. 2012;7:e39107. PubMed http://dx.doi.org/10.1371/journal.pone.0039107

28. Sullivan MJ, Petty NK, Beatson SA. Easyfig: a genome comparison visualizer. Bioinformatics. 2011;27:1009–10. PubMed http://dx.doi.org/10.1093/bioinformatics/btr039

29. Alikhan NF, Petty NK, Ben Zakour NL, Beatson SA. BLAST Ring Image Generator (BRIG): simple prokaryote genome comparisons. BMC Genomics. 2011;12:402. PubMed http://dx.doi.org/10.1186/1471-2164-12-402

30. Forde BM, Ben Zakour NL, Stanton-Cook M, Phan MD, Totsika M, Peters KM, et al. The complete genome sequence of *Escherichia coli* EC958: a high quality reference sequence for the globally disseminated multidrug resistant *E. coli* O25b:H4–ST131 clone. PLoS ONE. 2014;9:e104400. PubMed http://dx.doi.org/10.1371/journal.pone.0104400

31. Phan MD, Forde BM, Peters KM, Sarkar S, Hancock S, Stanton-Cook M, et al. Molecular characterization of a multidrug resistance IncF plasmid from the globally disseminated *Escherichia coli* ST131 clone. PLoS ONE. 2015;10:e0122369. PubMed http://dx.doi.org/10.1371/journal.pone.0122369

32. Andersen PS, Stegger M, Aziz M, Contente-Cuomo T, Gibbons HS, Keim P, et al. Complete genome sequence of the epidemic and highly virulent CTX-M-15–producing H30-Rx subclone of *Escherichia coli* ST131. Genome Announc. 2013;1:e00988-13. PubMed http://dx.doi.org/10.1128/genomeA.00988-13

33. Peirano G, van der Bij AK, Freeman JL, Poirel L, Nordmann P, Costello M, et al. Characteristics of *Escherichia coli* sequence type 131 isolates that produce extended-spectrum β-lactamases: global distribution of the *H*30-Rx sublineage. Antimicrob Agents Chemother. 2014;58:3762–7. PubMed http://dx.doi.org/10.1128/AAC.02428-14

34. Peirano G, Bradford PA, Kazmierczak KM, Badal RE, Hackel M, Hoban DJ, et al. Global incidence of carbapenemase-producing *Escherichia coli* ST131. Emerg Infect Dis. 2014;20:1928–31. PubMed http://dx.doi.org/10.3201/eid2011.141388

35. McGann P, Snesrud E, Ong AC, Appalla L, Koren M, Kwak YI, et al. War wound treatment complications due to transfer of an IncN plasmid harboring *bla*$_{OXA-181}$ from Morganella morganii to CTX-M-27–producing sequence type 131 *Escherichia coli*. Antimicrob Agents Chemother. 2015;59:3556–62. PubMed http://dx.doi.org/10.1128/AAC.04442-14

36. Kutumbaka KK, Han S, Mategko J, Nadala C, Buser GL, Cassidy MP, et al. Draft genome sequence of *bla*$_{NDM-1}$-positive *Escherichia coli* O25b-ST131 clone isolated from an environmental sample. Genome Announc. 2014;2:e00462-14. PubMed http://dx.doi.org/10.1128/genomeA.00462-14

Technical Appendix Table 1. Strain information, mapping, and assembly statistics

Strain	Year	Country (hospital, prefecture)	Location	Sample	*fimH* allele, *H*30Rx status	ESBL*	Mapping to EC958 genome		De novo assembly			Reference
							Sequencing depth	% Coverage	No. contigs	N50	Genome size	
KUN2145	2007	Japan (a, Kyoto)	Community	Blood	*H*22	CTX-M-14	36.3	96.0	201	77275	5261498	
KFEC6	2004	Japan (b, Kyoto)	Unknown	Unknown	*H*22	CTX-M-2	24.1	96.4	239	70346	5290551	
KSEC7	2002	Japan (c, Kyoto)	Unknown	Unknown	*H*30	CTX-M-14	26.0	98.5	187	69032	5165720	
KKEC3	2005	Japan (g, Shiga)	Unknown	Unknown	*H*30	TEM-12	24.9	97.7	174	71954	5128144	
KUN5823	2009	Japan (a, Kyoto)	Hospital	Urine	*H*30	TEM-132	39.9	96.3	103	153130	5093967	
JJ1886	2008	United States	Community	Blood	*H*30Rx	CTX-M-15	NA*	NA	6†	NA	5308284	(*32*)
KCH27	2009	Japan (c, Kyoto)	Unknown	Unknown	*H*30Rx	CTX-M-14	42.1	98.7	189	86306	5227333	
KUN3842	2008	Japan (a, Kyoto)	Community	Urine	*H*30Rx	CTX-M-15	38.3	98.6	153	84772	5216555	
SI48	2012	Japan (e, Shiga)	Unknown	Urine	*H*30Rx	CTX-M-15	37.0	98.4	171	101146	5253375	
ONEC14	2006	Japan (f, Shiga)	Unknown	Unknown	*H*30Rx	CTX-M-15	62.3	98.3	130	141652	5290408	
ONEC29	2007	Japan (f, Shiga)	Unknown	Unknown	*H*30Rx	CTX-M-15	40.7	98.6	176	87726	5151579	
KT6	2012	Japan (r, Kyoto)	Unknown	Urine	*H*30Rx	CTX-M-15	32.6	97.1	178	76197	5112138	
BRG23	2014	Japan (u, Osaka)	Hospital	Urine	*H*30Rx	CTX-M-15	55.8	97.4	151	125485	5151404	
KP14	2010	Japan (b, Kyoto)	Unknown	Urine	*H*30Rx	CTX-M-14+15	38.9	98.6	208	93237	5226221	
KS58	2011	Japan (c, Kyoto)	Unknown	Urine	*H*30Rx	CTX-M-15	40.6	98.6	179	70831	5140718	
EC958	2005	United Kingdom	Community	Urine	*H*30Rx	CTX-M-15	NA	NA	2‡	NA	5245369	(*30*)
KUN5191	2009	Japan (a, Kyoto)	Community	Urine	*H*30Rx	CTX-M-15	37.8	99.9	135	129047	5170127	
Ec 58	2009	Canada	Hospital	Blood	*H*30Rx	CTX-M-15	84.4	100.0	116	311639	5299942	(*33*)
Ec 31	2009	Canada	Healthcare-associated	Blood	*H*30Rx	CTX-M-15	86.8	100.0	94	282486	5300483	(*33*)
KS121	2012	Japan (c, Kyoto)	Unknown	Urine	*H*30Rx	CTX-M-15	34.7	98.0	216	75907	5346990	
BRG221	2014	Japan (c, Kyoto)	Community	Urine	*H*30Rx	CTX-M-14+15	36.5	96.7	197	87770	5233600	
KP75	2011	Japan (b, Kyoto)	Hospital	Blood	*H*30Rx	CTX-M-15	36.9	98.0	213	96467	5310621	
KP46	2010	Japan (b, Kyoto)	Unknown	Urine	*H*30Rx	CTX-M-15	37.5	98.0	204	91282	5315779	
BRG151	2014	Japan (i, Shiga)	Community	Urine	*H*30	Negative	32.8	97.8	151	94973	5134331	
BRG274	2014	Japan (w, Osaka)	Hospital	Urine	*H*30	Negative	45.4	96.9	179	95884	5187623	
BRG54	2014	Japan (t, Hyogo)	Community	Blood	*H*30	Negative	34.9	97.4	193	83029	5070132	
SNEC5	2003	Japan (h, Shiga)	Unknown	Unknown	*H*30	CTX-M-14	29.3	97.5	277	64189	5232935	
KUN4389	2009	Japan (a, Kyoto)	Hospital	Urine	*H*30	CTX-M-14	41.0	97.9	207	96127	5243970	
S100EC§	2009	Australia	Unknown	Rectal swab	*H*30	CTX-M-27	51.1	98.5	97	243020	5153420	(*3*)
USA 14	2008	United States	Community	Urine	*H*30	CTX-M-14	70.9	98.3	114	220820	5220386	(*33*)
BRG62	2014	Japan (t, Hyogo)	Community	Urine	*H*30	CTX-M-14	52.0	98.9	159	102298	5158745	
KS46	2011	Japan (c, Kyoto)	Unknown	Urine	*H*30	CTX-M-14	40.8	97.7	207	84629	5116558	
KUN3273	2008	Japan (a, Kyoto)	Community	Urine	*H*30	CTX-M-14	38.6	97.6	181	105906	5128255	
ONEC7	2006	Japan (f, Shiga)	Unknown	Unknown	*H*30	CTX-M-14	33.3	98.1	220	80399	5196172	
KN94	2012	Japan (d, Kyoto)	Unknown	Urine	*H*30	CTX-M-14	48.8	97.5	131	119924	5223561	
KT37	2012	Japan (r, Kyoto)	Unknown	Urine	*H*30	CTX-M-14	24.2	97.8	289	47475	5125852	
S135EC§	2005	Canada	Community	Blood	*H*30	CTX-M-14	58.9	98.8	116	152032	5275688	(*3*)
FR 11	2008	France	Community	Urine	*H*30	CTX-M-14	36.7	97.6	106	169392	5131354	(*33*)
Ec# 584	2011	Vietnam	Unknown	Intraabdominal	*H*30	CTX-M-27	48.4	98.1	346	75552	5412329	(*34*)
ECNZ 35	2010	New Zealand	Hospital	Blood	*H*30	CTX-M-14	63.9	98.4	115	159997	5309674	(*33*)
EcSA01	2008	South Africa	Community	Urine	*H*30	CTX-M-14	68.3	97.5	80	194881	5198654	(*33*)
Ec 32	2009	Canada	Community	Blood	*H*30	CTX-M-14	61.5	98.3	174	184369	5352796	(*33*)

Strain	Year	Country (hospital, prefecture)	Location	Sample	*fimH* allele, *H*30Rx status	ESBL*	Mapping to EC958 genome: Sequencing depth	Mapping to EC958 genome: % Coverage	De novo assembly: No. contigs	De novo assembly: N50	De novo assembly: Genome size	Reference
KUN8768	2011	Japan (a, Kyoto)	Community	Urine	*H*30	CTX-M-27	35.5	97.5	170	93073	5184482	
SN37	2010	Japan (h, Shiga)	Unknown	Sputum	*H*30	CTX-M-27	35.3	96.3	164	79492	5039880	
SI43	2012	Japan (e, Shiga)	Unknown	Urine	*H*30	CTX-M-27	38.5	97.2	171	82979	5017470	
KT10	2012	Japan (r, Kyoto)	Unknown	Blood	*H*30	CTX-M-27	53.3	95.0	119	133403	4954097	
KUN3594	2008	Japan (a, Kyoto)	Hospital	Urine	*H*30	CTX-M-27	35.8	97.1	124	93754	5021116	
KFEC8	2004	Japan (b, Kyoto)	Unknown	Unknown	*H*30	CTX-M-27	32.2	97.1	235	55958	5097618	
S107EC§	2010	Australia	Unknown	Urine	*H*30	CTX-M-27	46.8	97.1	97	191225	5092957	(*3*)
S108EC§	2009	Australia	Unknown	Blood	*H*30	CTX-M-27	61.9	97.4	95	192487	5121514	(*3*)
KSEC29	2006	Japan (c, Kyoto)	Unknown	Unknown	*H*30	CTX-M-27	39.3	97.3	138	102764	5064236	
KN1	2010	Japan (d, Kyoto)	Unknown	Urine	*H*30	CTX-M-27	46.2	97.3	141	120021	5179897	
ONEC27	2007	Japan (f, Shiga)	Unknown	Unknown	*H*30	CTX-M-27	37.8	97.2	190	76207	5046307	
BRG120	2014	Japan (s, Aichi)	Hospital	Sputum	*H*30	CTX-M-27	41.3	97.6	124	140794	5094906	
SN65	2011	Japan (h, Shiga)	Unknown	Pus	*H*30	CTX-M-27	47.0	98.5	148	124167	5105443	
EcAZ 156	2013	Thailand	Unknown	Urine	*H*30	CTX-M-27	54.0	97.2	128	159590	5130003	(*34*)
KS26	2010	Japan (c, Kyoto)	Unknown	Urine	*H*30	CTX-M-27	51.3	97.2	162	104636	5105370	
MRSN17749¶	2013	United States	Hospital	Groin swab	*H*30	CTX-M-27	NA	NA	92	191197	5046460	(*35*)
IEH71520¶	2014	United States	House environment	Vacuum cleaner dust	*H*30	CTX-M-27	NA	NA	202	67135	5153432	(*36*)
Ec 24	2008	Canada	Hospital	Blood	*H*30	CTX-M-27	84.9	97.3	77	216849	5077997	(*33*)
KUN5781	2009	Japan (a, Kyoto)	Hospital	Blood	*H*30	CTX-M-27	36.3	97.2	147	116898	5066641	

*ESBL, extended-spectrum β-lactamase; NA, not applicable.
†Chromosome and 5 plasmids.
‡Chromosome and 1 plasmid.
§Short reads were mapped and assembled using the same methods as our sequenced isolates.
¶Draft genome.

Technical Appendix Table 2. Genetic structures that flank ESBL genes*

ESBL type: group, subtype	Genetic structure				Clade in the tree or group, number of isolates			
					C/*H*30R			
					C1/*H*30R			
	Upstream (bp)	Downstream (bp)	Type in Figure 1 and Technical Appendix Figure 2†	GenBank accession no.	C1-M27, n = 19	Others, n = 16	C2/*H*30Rx, n = 18	Other than C/*H*30R, n = 5
$bla_{CTX-M-9}$ group								
$bla_{CTX-M-27}$	IS*26*-ΔIS*Ecp1* (208)	ΔIS*903D* (391)-IS*26*	9a2, 9a2′‡	AB976590	18[b]			
	IS*26*-ΔIS*Ecp1* (208)	ΔIS*903D* (226)-IS*26*	9a3	AB985520	1			
	IS*26*-ΔIS*Ecp1* (388)	IS*903D*	9e1	LC091535§		2		
$bla_{CTX-M-14}$	IS*Ecp1*	ΔIS*903D*	9d3, 9d3′¶, 9d3″#	AB976599, LC091534¶, LC107627[#]		6¶#	2**	2
	IS*Ecp1*	IS*903D*	9d1	AB976598		4	1[g]	
	ΔIS*Ecp1*	ΔIS*903D*	9d2, 9d2′‡‡	AB976605		2		
	ΔIS*Ecp1*	IS*903D*	9d4′§§	AB976604		2		
$bla_{CTX-M-1}$: $bla_{CTX-M-15}$	IS*Ecp1*	*orf477*	1a1	AB976566			9**	
	IS*26*-ΔIS*Ecp1* (497)	*orf477*	1b	AB976569			4††	
	IS*26*-ΔIS*Ecp1* (24)	*orf477*	1c, 1c′¶¶	AB976574, LC107628[j]			4	
$bla_{CTX-M-2}$: $bla_{CTX-M-2}$	IS*Ecp1*	Downstream bla_{KLUA}##	2a1	AB976588				1
bla_{TEM}								
bla_{TEM-12}	Tn*2*	Tn*2*	T1	LC091536§				1
$bla_{TEM-132}$	Tn*2*	Tn*2*	T2	LC091537§				1

*ESBL, extended-spectrum β-lactamase.
†The classification and numbering of the structures follows these in our previous publication (*26*).
‡One isolate from another study (MRSN17749) had a contig of ΔIS*Ecp1* (208bp)-$bla_{CTX-M-27}$-ΔIS*903D* (391bp) without the IS*26* flanking structure. However, the lengths of the truncated IS*Ecp1* and IS*903D* structures suggest the isolate had the 9a2 structure.
§New sequence found in this study (no identical sequence deposited in GenBank).
¶One isolate (BRG62) had the 9d3′ structure, a variant of 9d3. The only difference between 9d3 and 9d3□ is one nucleotide (1 aa) change in *tnpA* of ΔIS*903D*.
#One isolate (ECNZ 35) had the 9d3” structure, a variant of 9d3. The 9d3” structure has 1 nt change (synonymous substitution) in $bla_{CTX-M-14}$. The isolate may have IS*Ecp1*-$bla_{CTX-M-14}$-IS*903D* structure because the $bla_{CTX-M-14}$-containing contig included 5′ truncated IS*903D* but remaining sequence of IS*903D* was found in another contig.
**One isolate was positive for both 9d1 and 1b.
††One isolate was positive for both 9d3 and 1a1.
‡‡One isolate (S135EC) may have IS*Ecp1*-$bla_{CTX-M-14}$-ΔIS*903D* structure because the $bla_{CTX-M-14}$-containing contig included 3′ truncated IS*Ecp1* but remaining sequence of IS*Ecp1* was found in another contig.
§§These 2 isolates (USA 14 and EcSA01) may have IS*Ecp1*-$bla_{CTX-M-14}$-IS*903D* (9d1) structure because the $bla_{CTX-M-14}$-containing contig included 5′ truncated IS*903D* but remaining sequence of IS*903D* was found in another contig.
¶¶One isolate (BRG62) had the 9d3” structure, a variant of 9d3. The only difference between 9d3 and 9d3” is one nucleotide (1 aa) change in *tnpA* of ΔIS*903D*.
##The nucleotide sequence was identical to the region between *kluA-1* and *orf3* of *Kluyvera ascorbata* (GenBank accession no. AJ272538).

Technical Appendix Figure 1.

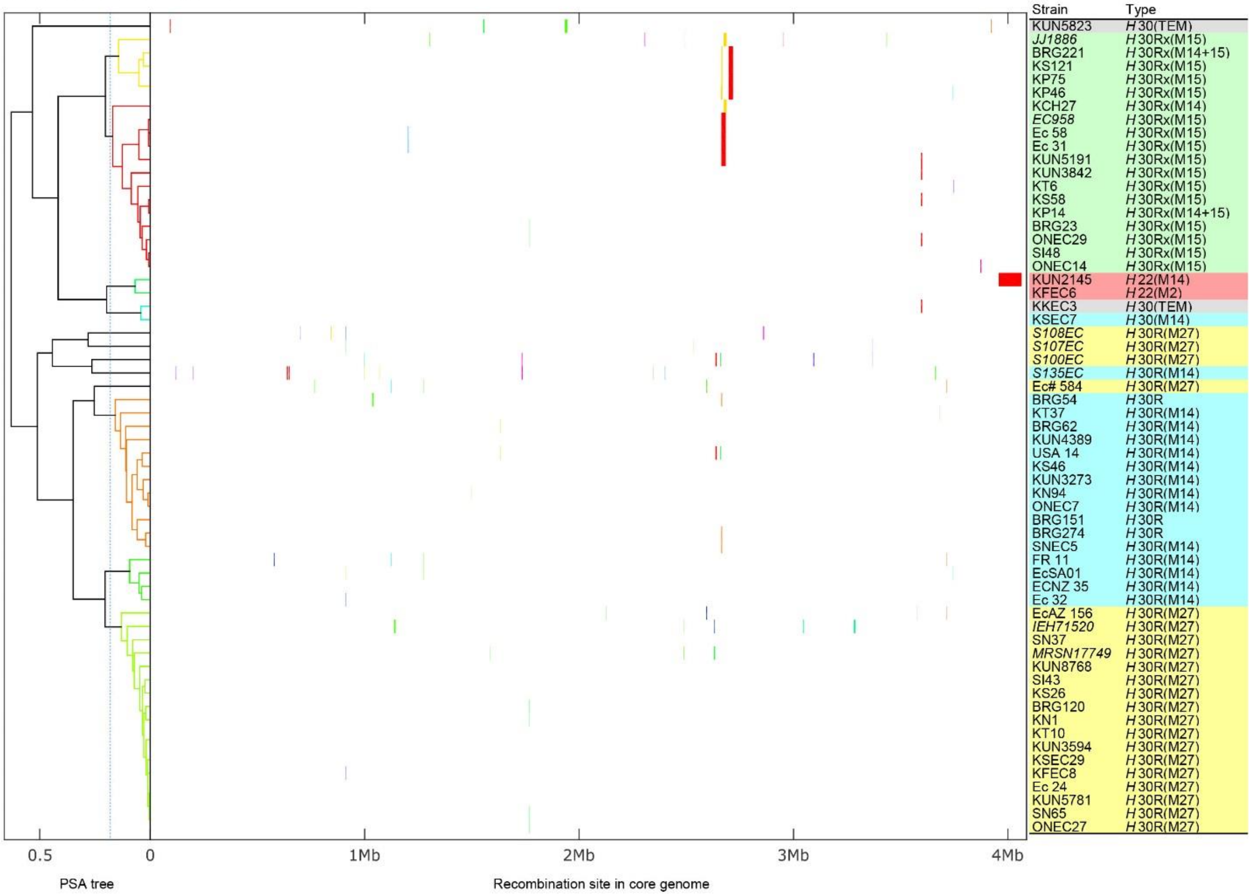

Technical Appendix Figure 1. Recombinant regions identified by BRATNextGen. The same core genome used for construction of the single-nucleotide polymorphism–based phylogenetic tree (Figure 1 in main text) was used for the analysis. The tree in the left is a proportion of shared ancestry tree. A cutoff value of 0.15 was chosen to form clusters of the C1-M27 clade, C1/*H*30R isolates other than those of the C1-M27 clade, and C2/*H*30Rx clade. The strain names and types are colored as same as those in Figure 1. ESBL types are indicated in parentheses of Type column. The middle panel shows a horizontal representation of the recombinant segments using color bars. Segments of the same color and the same column derived from the same origin. A total of 79 segments (304,782 bp) including 3,453 SNPs were associated with recombination.

Technical Appendix Figure 2.

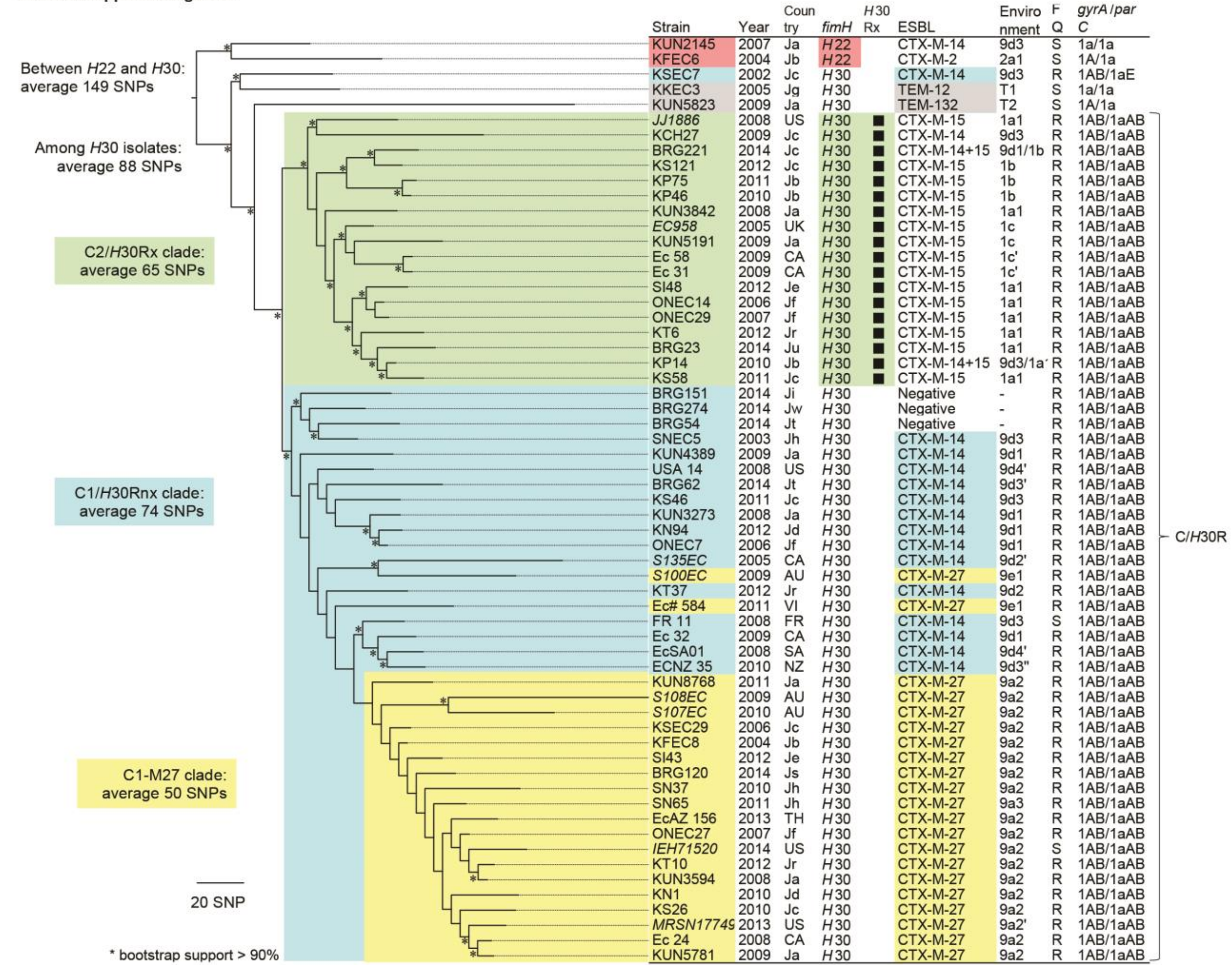

Technical Appendix Figure 2. Phylogenetic tree build from recombination-free core genome. This maximum-likelihood phylogram is based on a 3,781,868-bp recombination-free core genome and a total of 1,827 single-nucleotide polymorphisms. The tree is rooted by using the outgroup *H*22 isolates and asterisks indicates bootstrap support >90% from 100 replicates. The clustering results were as same as the tree built from the whole core genome shown in Figure 1. The ciprofloxacin-resistant C/*H*30R cluster comprised the C2/*H*30Rx and C1/*H*30R clades. All of the *H*30Rx isolates belonged to the C2/*H*30Rx clade. The C1/*H*30R clade included CTX-M-14-produing *H*30R, non-ESBL–producing *H*30R, and CTX-M-27–producing *H*30R isolates. CTX-M-27–producing isolates belonged to the C1-M27 clade within the C1/*H*30R clade except two isolates (S100EC and Ec #584). The bootstrap value for the root of the M27 clade was 76%.

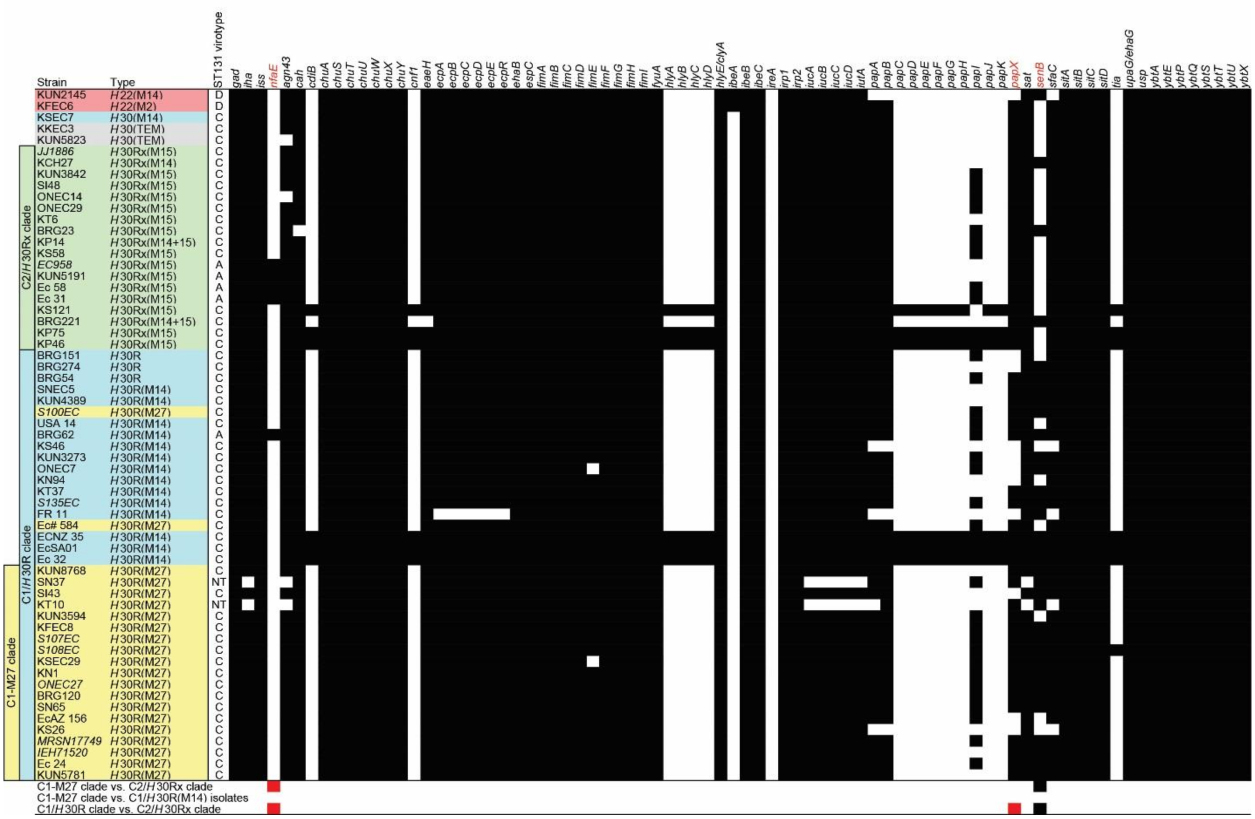

Technical Appendix Figure 3. *Escherichia coli* sequence type (ST) 131 virotypes and virulence genes. Black squares indicate presence of each gene. Results of statistical tests for gene prevalence comparison between clades are shown at the bottom rows; black indicates high prevalence of the former clade, and red indicates high prevalence of the latter clade. ST131 virotype C was prevalent in common. Virotype NT indicates nontypeable. The C1-M27 clade isolates more frequently had *senB* enterotoxin gene than C2/*H*30Rx isolates but the other C1/*H*30R isolates also frequently had it. Two genes (*nfaE* and *papX*) were prevalent in the C2/*H*30Rx clade than the C1/*H*30R clade.

Technical Appendix Figure 3.

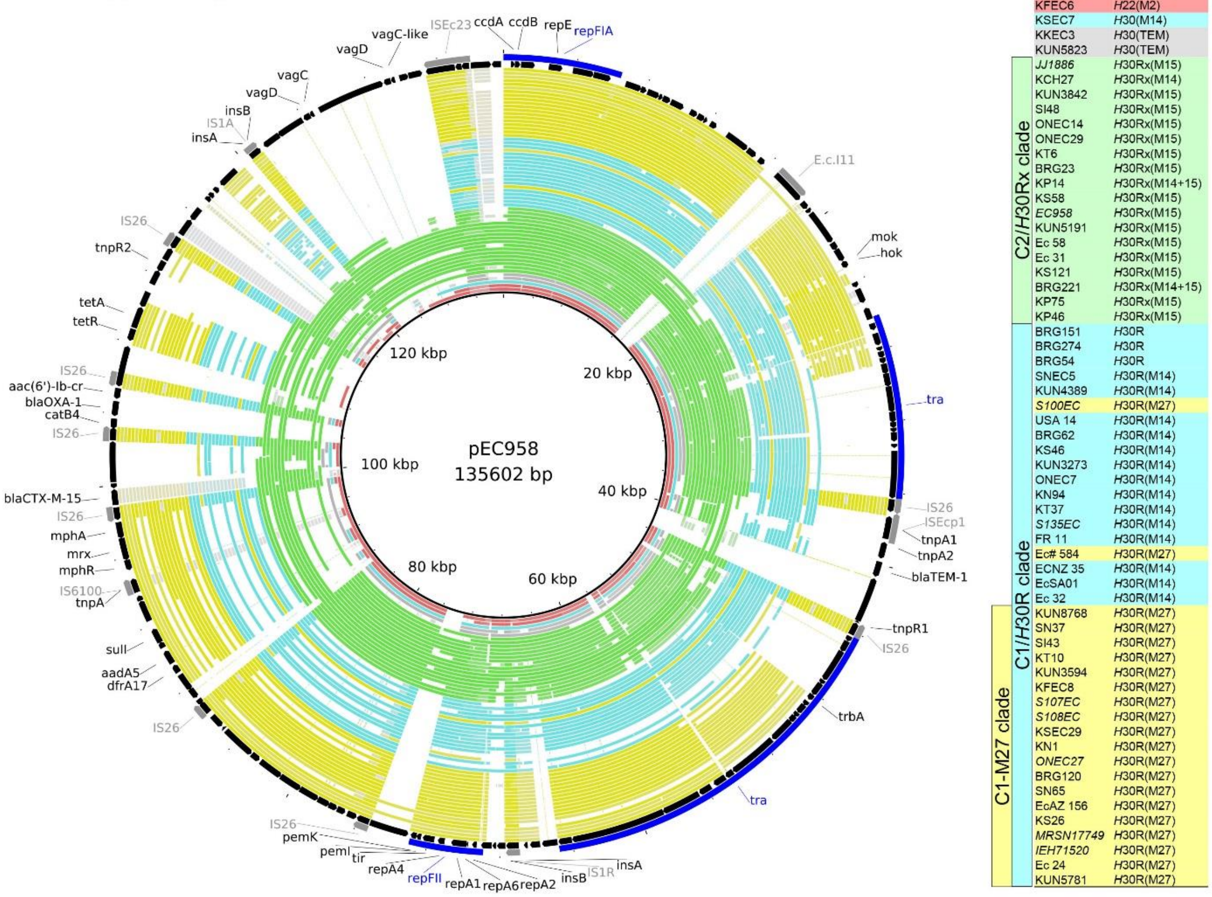

Technical Appendix Figure 4. Comparison of genomes of *Escherichia coli* sequence type (ST) 131 isolates with the pEC958 plasmid of CTX-M-15–producing ST131 C2/*H*30Rx reference strain EC958. Rings drawn by BLAST Ring Image Generator show the presence of the pEC958-like regions and colored according to colors in Figure 1. Colored segments indicate >90% similarity, and gray segments indicate >70% similarity by BLAST comparison between the regions of interest and each genome. The M27 clade lacked the first part of the transfer regions (*tra*). Some regions common to both C2/*H*30Rx and C1/*H*30R clades are present, but the presence or absence of other regions are divergent even within the same clade. The presence of resistance genes is also shown in Technical Appendix Figure 5.

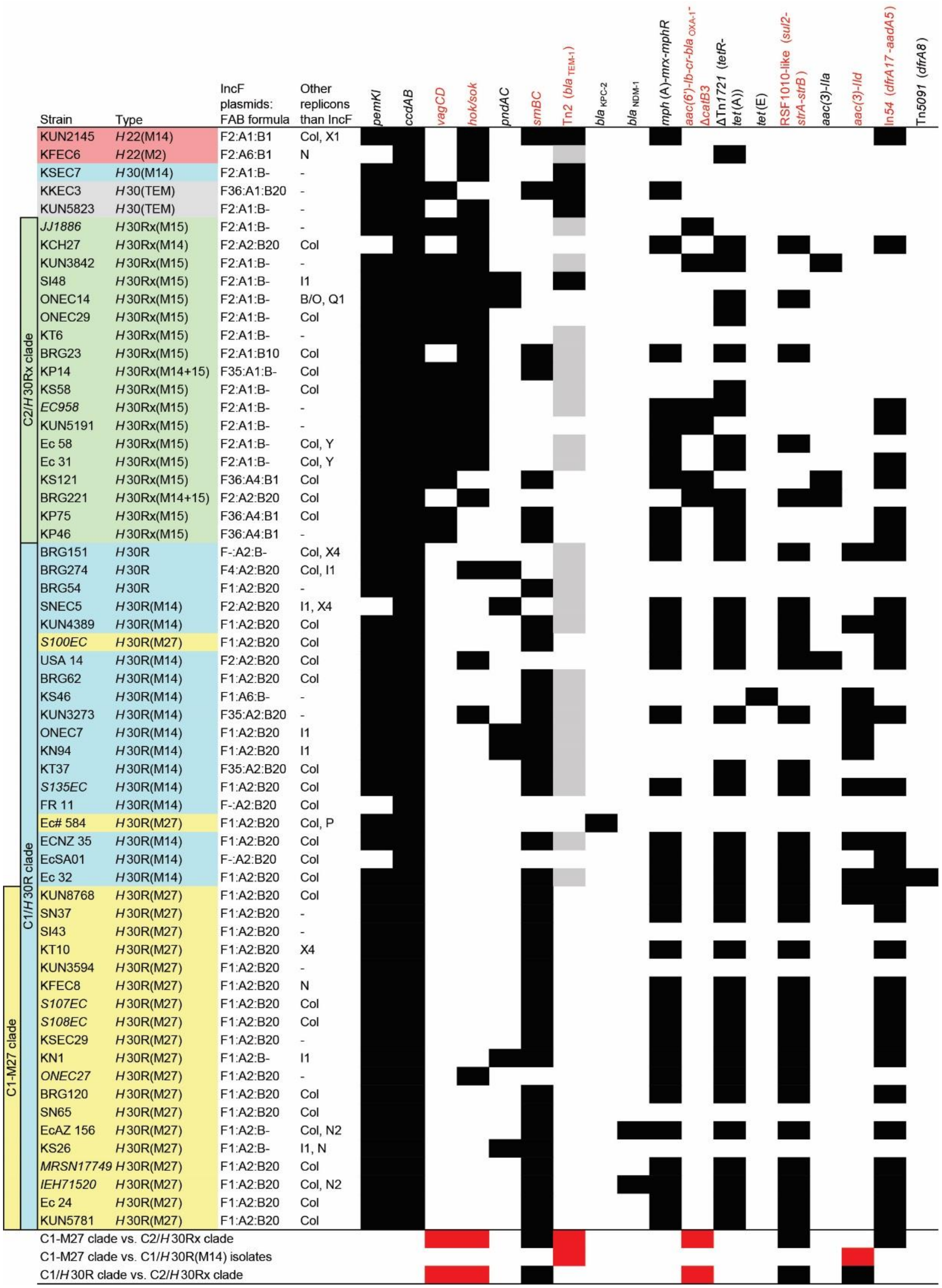

Technical Appendix Figure 5. Plasmid replicons, plasmid addiction systems, and antimicrobial resistance genes of extraintestinal pathogenic *Escherichia coli*. Black indicates presence of each gene. Gray area of Tn*2* column indicates truncated Tn*2*. Results of statistical tests for gene prevalence comparison between

clades are shown at the bottom rows; black indicates high prevalence of the former clade and red indicates high prevalence of the latter clade. F1:A2:B20 IncF plasmids were prevalent in the C1/*H*30R clade while F2:A1:B- plasmids were prevalent in the C2/*H*30Rx clade. Three CTX-M-14–producing C2/*H*30Rx isolates had mixture types of replicons from the CTX-M-15–producing C2/*H*30Rx and CTX-M-14–produing C1/*H*30R isolates. Only C2/*H*30Rx isolates had *vagCD* plasmid addiction system, *aac(6')-Ib-cr-*$bla_{OXA\text{-}1}$-Δ*catB3* resistance gene set. C2/*H*30Rx isolates more frequently had *vagCD* and *hok/sok* plasmid addiction systems than the C1/*H*30R isolates. *srnBC* plasmid addiction system and *sul2-strA-strB* resistance gene set originally found in RSF1010 plasmid were more frequently found in the C1/*H*30R isolates than the C2/*H*30Rx isolates. None of the C1-M27 clade isolates had Tn*2* ($bla_{TEM\text{-}1}$). Class 1 integron In*54* (*dfrA17-aadA5*) was more frequently found in the C1-M27 clade isolates than the C2/*H*30Rx isolates. CTX-M-14–producing C1/*H*30R isolates more frequently had *aac(3)-IId* than the C1-M27 clade or C2/*H*30Rx isolates. Two C1-M27 isolates carried $bla_{NDM\text{-}1}$ on IncN2 plasmid backbone and ΔIS*Aba125*-IS*Ec33*-Δ*ISAba125*-$bla_{NDM\text{-}1}$-ble_{MBL}-Δ*trpF*-IS*Sen4*-Tn*5403* structure and 1 CTX-M-14–producing C1/*H*30R isolate had $bla_{NDM\text{-}1}$ in IS*Kpn27*-Δ$bla_{TEM\text{-}1}$-$bla_{KPC\text{-}2}$-Δ*traN-korC-klcA* structure.
